# Bevacizumab in HER2-negative inflammatory breast cancer

**DOI:** 10.18632/oncoscience.324

**Published:** 2016-11-15

**Authors:** François Bertucci, Anthony Goncalves, Patrice Viens

**Affiliations:** Department of Medical Oncology, Centre de Recherche en Cancérologie de Marseille, Institut Paoli-Calmettes, Aix-Marseille University, Marseille, France

**Keywords:** bevacizumab, inflammatory breast cancer, pathological complete response, survival, VEGF

Inflammatory breast cancer (IBC) is a rare (∼5%) but aggressive form of breast cancer with high metastatic potential [1]. Despite the successful introduction of neoadjuvant anthracycline/taxane-based chemotherapy, combined with neoadjuvant/adjuvant trastuzumab in case of HER2-positivity and adjuvant hormone therapy in case of hormone receptor (HR)-positivity, the 5-year survival ranges from 50 to 60% as compared to more than 85% in non-IBC. Besides its aggressiveness and frequent resistance to treatment, IBC displays other characteristics that make progresses difficult. The disease is rare and the diagnosis challenging, based on clinical signs including rapid (no more than 6 months) onset of breast erythema and oedema, with or without underlying palpable mass. Such features exclude IBC from mass screening, lead to frequent misdiagnosis and delayed diagnosis, and complicate the set-up of IBC-specific clinical trials. However, IBC is biologically different from non-IBC [2]. Notably, IBCs are more angiogenic tumours than non-IBC, displaying higher microvessel density, and showing presence of dermal lymphovascular tumour emboli. Combined with the negative prognostic value of VEGF expression, these observations made IBC attractive for testing anti-angiogenic drugs.

Based on these observations, and with the intent to continue to study IBC as a separate entity as we did previously [3, 4], we launched in 2008 the BEVERLY-1 trial, a French, multicentric, single arm, prospective phase 2 trial assessing the benefit of neoadjuvant/adjuvant bevacizumab in patients with HER2-negative (BEVERLY-1) non-metastatic IBC. Bevacizumab is a monoclonal antibody that inhibits tumour angiogenesis by targeting VEGF, approved in 2008 by the US Food and Drug Administration under an accelerated plan in combination with chemotherapy in metastatic breast cancer. During the neo-adjuvant phase, the patients received four cycles of FEC100 plus bevacizumab every three weeks, followed by four cycles of docetaxel plus bevacizumab every three weeks. Then, the surgery included total mastectomy and axillary lymph node dissection, and was followed by adjuvant radiation therapy plus 10 cycles of bevacizumab (every 3 weeks), and hormone therapy if the tumour was HR-positive. Each patient theoretically received 8 cycles of neo-adjuvant chemotherapy and 18 cycles of neo-adjuvant/adjuvant bevacizumab. The primary endpoint was the pathological complete response (pCR) rate in breast and axillary lymph nodes after neo-adjuvant treatment, centrally assessed using the Sataloff classification: the regimen was regarded as efficacious if 30% or more patients had a pCR. Secondary endpoints included disease-free survival (DFS), overall survival (OS), safety, and analysis of circulating tumour cells and endothelial cells. BEVERLY-1 was the first prospective clinical trial testing the addition of neoadjuvant/adjuvant bevacizumab in patients with HER2-negative IBC. The main efficacy results can be summarised and discussed as follows [5].

Nineteen of 100 patients enrolled in BEVERLY-1 achieved centrally defined pCR (19%, 95%CI 11.84-28.07). Although established in a non-randomised study, this disappointing rate does suggest that addition of bevacizumab does not increase the pCR rate. It is inferior or similar to rates reported in IBC without bevacizumab after high-dose anthracycline-based chemotherapy: 32% and 20% in the Pegase 02 [4] and 07 [3] trials. In five recent randomised trials (GeparQuinto, NSABP-B40, ARTemis, CALGB 40603, S0800) dedicated to HER2-negative non-IBC, bevacizumab addition to neoadjuvant anthracycline/taxane-based chemotherapy significantly increased the pCR rate. In two trials (ARTemis, S0800), an exploratory analysis of IBC patients showed no significant advantage for bevacizumab, although the number of samples was small. Potential factors responsible for the disappointing pCR rate in IBC when compared with non-IBC include reduced chemosensitivity of IBC perhaps in part related to the role of breast cancer stem cells (CSCs) in IBC, increase of the tumour population of classically chemoresistant breast CSCs due to the hypoxia generated by bevacizumab [6], and more prominent roles of angiogenesis, but also of lymphangiogenesis and vasculogenesis [7] that should make insufficient the mere blocking of VEGF by bevacizumab in IBC.

With a median follow-up of 45 months, the 3-year DFS was 57% (95%CI 47-66) and the 3-year OS was 75% (95%CI 65-83), close to those reported (∼60% and ∼80% respectively) without bevacizumab in the recent Pegase 07 trial, which tested the benefit of adding adjuvant docetaxel-5-fluorouracil regimen after neoadjuvant dose-intense anthracycline-based regimen [3]. Even if additional follow-up, planned at 5 years, is required, our results, compared with literature, do not suggest survival benefit with bevacizumab in HER2-negative IBC, as reported in the recent randomized trials testing adjuvant or neoadjuvant bevacizumab in HER2-negative non-IBC. Potential explanations for these poor results include increase of invasive properties of breast cancer xenografts after anti-angiogenic treatments [8], increase of breast CSCs during bevacizumab [6], and a possible rebound of tumour cell growth after completion of therapy [9].

In conclusion, our results suggest that bevacizumab addition does not provide benefit in the whole population of patients with non-metastatic HER2-negative IBC. In this context, it seems difficult to attribute to bevacizumab the high pCR rate (63.5%) observed in the BEVERLY-2 trial [10], similarly designed, conducted in parallel but dedicated to HER2-positive IBC. Clearly, longer follow-up and correlative studies for identifying predictors for benefit are needed, as well as meta-analyses of trials including IBC patients. However, besides the results, BEVERLY-1 highlights a very important point for the future of IBC research: the possibility of a relatively high and quick patients' enrolment, with 100 patients enrolled over a 21-month period despite the rarity of disease. Similar high enrolment (52 patients over 12 months) had been observed in BEVERLY-2 [10]. These figures should encourage us to set up international randomized clinical trials dedicated to this “orphan” and always so mysterious disease. These trials should include translational research to improve our molecular understanding of disease and of bevacizumab's mechanisms of action and resistance, and to identify patients who stand to benefit from treatment.
